# Structure of catabolite activator protein with cobalt(II) and sulfate

**DOI:** 10.1107/S2053230X14005366

**Published:** 2014-04-15

**Authors:** Ramya R. Rao, Catherine L. Lawson

**Affiliations:** a Department of Chemistry and Chemical Biology, Rutgers University, Piscataway, NJ 08854, USA

**Keywords:** catabolite activator protein, *Escherichia coli*

## Abstract

The crystal structure of cyclic AMP–catabolite activator protein (CAP) from *Escherichia coli* containing cobalt(II) chloride and ammonium sulfate is reported at 1.97 Å resolution. Each of the two CAP subunits in the asymmetric unit binds one cobalt(II) ion, in each case coordinated by N-terminal domain residues His19, His21 and Glu96 plus an additional acidic residue contributed *via* a crystal contact. The three identified N-terminal domain cobalt-binding residues are part of a region of CAP that is important for transcription activation at class II CAP-dependent promoters. Sulfate anions mediate additional crystal lattice contacts and occupy sites corresponding to DNA backbone phosphate positions in CAP–DNA complex structures.

## Abstract 2

The crystal structure of *E. coli* catabolite activator protein with bound cobalt(II) and sulfate ions at 1.97 Å resolution is reported.

## 1. Introduction   

CAP (catabolite activator protein) is a DNA-binding protein and transcription activator that recruits RNA polymerase to more than 100 genes in *Escherichia coli* (Busby & Ebright, 1999 ▶; Lawson *et al.*, 2004 ▶; Zheng *et al.*, 2004 ▶). The CAP dimer consists of two identical 209-residue subunits, each with an N-terminal binding domain for allosteric effector cAMP, and a C-terminal winged helix–turn–helix DNA-binding motif. CAP has three defined ‘activating regions’, patches of surface-exposed residues that interact with RNA polymerase (RNAP) in specific CAP-dependent promoter classes. Activating region 1 (AR1), located on the C-terminal domain, attracts a flexibly tethered RNAP α subunit C-terminal domain to bind to a DNA site adjacent to CAP (Benoff *et al.*, 2002 ▶; Lawson *et al.*, 2004 ▶; Hudson *et al.*, 2009 ▶). AR2, located on the N-terminal cAMP-binding domain, functions specifically at class II promoters and is predicted to make direct contact with an acidic loop of the RNAP α^I^ subunit N-terminal domain (αNTD^I^; Niu *et al.*, 1996 ▶; Busby & Ebright, 1999 ▶; Lawson *et al.*, 2004 ▶). AR3 is also located on the N-terminal domain and is predicted to make direct contact with RNAP σ subunit region 4 (Rhodius & Busby, 2000 ▶).

Three distinct crystal forms of native cAMP-bound CAP from *E. coli* have been reported previously: (i) space group *P*2_1_2_1_2_1_ with one dimer in the asymmetric unit (PDB entry 1g6n; McKay *et al.*, 1982 ▶; Passner *et al.*, 2000 ▶), (ii) space group *P*2_1_ with one dimer in the asymmetric unit (PDB entry 4hzf; Rodgers *et al.*, 2013 ▶) and (iii) space group *P*1 with two dimers in the asymmetric unit (PDB entry 2gzw; RIKEN Structural Genomics/Proteomics Initiative, unpublished work). We describe here the structure of cAMP–CAP in a novel fourth crystal form (space group *P*2_1_2_1_2 with one dimer in the asymmetric unit) in which cobalt(II) binds to key residues of CAP AR2. In addition, discrete sulfate-anion-binding sites are found near AR2, near the cAMP-binding site and at positions analogous to DNA phosphate positions in CAP–DNA complexes. This crystal form of cAMP–CAP was fortuitously obtained during efforts to structurally characterize a 2:1 CAP dimer–DNA complex under conditions that apparently disrupted the protein–DNA interaction (Rao, 2011 ▶).

## 2. Materials and methods   

### 2.1. Preparation of CAP   

Full-length CAP (209 residues) was expressed and purified by cAMP-affinity chromatography, as described in Zhang *et al.* (1991 ▶), and further purified on a Heparin 16/10 FF column (GE Healthcare). CAP was then concentrated and buffer-exchanged to approximately 360 µ*M* dimer (approximately 18 mg ml^−1^) in 20 m*M* Tris pH 8.0, 0.1 m*M* EDTA, 1 m*M* DTT, 200 m*M* NaCl, 0.02%(*w*/*v*) NaN_3_. The protein concentration was determined using a Bradford assay. CAP purity was estimated to be >95%.

### 2.2. Crystallization and data collection   

A 71 bp DNA duplex containing two tandem CAP sites separated by 32 base pairs was designed according to a promoter sequence described by Tebbutt *et al.* (2002 ▶) and was assembled using three oligonucleotides (Rao, 2011 ▶, p. 11). DNA oligonucleotides were obtained from IDT and were annealed to form duplex DNA using a standard laboratory procedure (Locasale *et al.*, 2009 ▶). The CAP–DNA complex used in crystallization trials consisted of 0.1 m*M* CAP dimer, 0.05 m*M* DNA duplex, 0.4 m*M* cyclic AMP.

Crystallization screening was by hanging-drop vapor diffusion using Hampton Research crystal screens and pre-greased VDX plates. Optimization of Crystal Screen 2 condition No. 25 yielded ruby-red-colored crystals that appeared within 3 d at 20°C in 0.01 *M* CoCl_2_.6H_2_O, 0.1 *M* MES monohydrate pH 6.5, 2.5 *M* ammonium sulfate.

Crystals were mounted on nylon loops and flash-cooled in liquid nitrogen after being submerged briefly in a cryoprotectant solution comprised of 66.5%(*w*/*w*) Paratone-N, 28.5%(*w*/*w*) paraffin oil, 5%(*w*/*w*) glycerol. X-ray diffraction data were collected on beamline X8C at the National Synchrotron Light Source at Brookhaven National Laboratory to 1.97 Å resolution.

### 2.3. Structure determination and refinement   

The diffraction data were processed and scaled assuming primitive orthorhombic lattice symmetry using the *HKL*-2000 package (Otwinowski & Minor, 1997 ▶). Molecular-replacement search models were prepared using selected coordinates from PDB entry 1zrc (Napoli *et al.*, 2006 ▶). Molecular-replacement trials were carried out in all possible primitive orthorhombic space groups using *Phaser* (McCoy *et al.*, 2007 ▶). An unambiguous solution was obtained. The N-terminal domain dimer of CAP (residues 1–136) was positioned first, yielding a *Z*-score of 18.7 in space group *P*22_1_2_1_; the next highest *Z*-score was 10.0 in space group *P*22_1_2. The two C-terminal domains (residues 137–209) were located in subsequent *Phaser* runs. After the molecular replacement, the model coordinates and diffraction data *hkl* Miller indices were transformed to the standard *P*2_1_2_1_2 setting.

Noncrystallographic twofold symmetry observed in the self-rotation function and Matthews coefficient analysis (Kantardjieff & Rupp, 2003 ▶; Matthews, 1968 ▶) provided additional support for the assignment of one complete CAP dimer in the crystal asymmetric unit. Upon inspection of crystal packing and likelihood-weighted electron density, it became evident that the crystal structure did not contain DNA.

Crystallographic refinement was initially performed using *REFMAC*5 (Murshudov *et al.*, 2011 ▶) and in the final stages using *PHENIX* (Adams *et al.*, 2010 ▶). Noncrystallographic symmetry restraints for CAP N-terminal and C-terminal domains were maintained and separately defined. The model was inspected between refinement runs and manually adjusted to fit likelihood-weighted electron-density maps using *Coot* (Emsley & Cowtan, 2004 ▶). Solvent atoms were initially added manually using conservative criteria; in the final refinement stages, the *phenix.refine* water-picking protocol was used with default settings. Cobalt(II) and sulfate ligands were manually placed in positions of strong electron density at the protein surface that could not be satisfactorily modeled as solvent owing to density strength, shape and local stereochemistry criteria.

### 2.4. Structure analysis   

The quality and stereochemistry of the model and fit to electron density were evaluated using validation tools in *Coot*. The *CCP*4 suite (Winn *et al.*, 2011 ▶) programs *CONTACT* and *SUPERPOSE* were used to evaluate contacts between protein and ligands and to perform superposition calculations. Molecular-graphics images were created using *UCSF Chimera* (Pettersen *et al.*, 2004 ▶).

## 3. Results   

**Figure 1:**
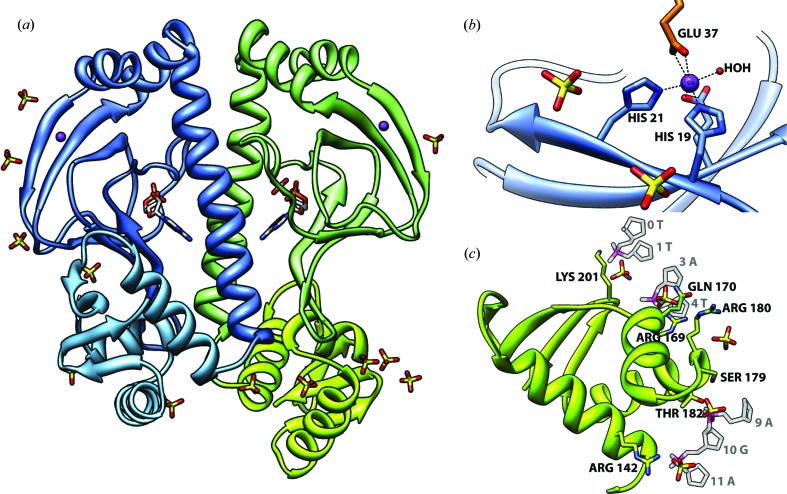
cAMP–CAP dimer with bound cobalt(II) and sulfate ligands (CAP subunits, blue/light-blue and green/lime ribbons; cAMP and sulfate, stick representation with atom-type colors; Co^2+^, purple spheres). (*a*) Crystal asymmetric unit composed of one cAMP–CAP dimer, two Co^2+^ ions and 13 sulfate ligands. (*b*) Co^2+^ coordination in subunit *A* (crystal lattice neighbor shown at top in orange). (*c*) C-terminal domain of subunit *B* shown with bound sulfates and selected DNA sugar-phosphate backbone fragments from a superimposed CAP–DNA structure [PDB entry 1zrc (Napoli *et al.*, 2006 ▶), translucent grey/pink sticks]. DNA numbering follows Fig. 1 ▶ of Lawson *et al.* (2004 ▶).

Our efforts to characterize a CAP–DNA complex fortuitously yielded a crystal structure for cAMP–CAP without DNA in a novel crystal form in which cobalt and sulfate ligands are bound to CAP. This structure is shown schematically in Fig. 1 ▶(*a*). The crystal asymmetric unit contains one CAP dimer (residues 7–207 of chain *A* and 7–206 of chain *B* were modeled out of 210 total residues per subunit), with cAMP ligands bound as expected within the N-terminal domain (Berman *et al.*, 2005 ▶), two cobalt(II) cations, 13 sulfate anions and 420 solvent atoms.

The main-chain torsion angles of most residues (96%) lie in preferred regions of the Ramachandran plot, with the remainder (4%) in the allowed regions. The final *R* factor is 19.4% and *R*_free_ is 22.9% (Table 1 ▶).

The conformation of the CAP dimer in this new crystal form is essentially identical to the conformation observed in earlier structure reports for cAMP–CAP (McKay *et al.*, 1982 ▶; Passner *et al.*, 2000 ▶), with two cAMP-binding N-terminal domains related by local twofold rotational (*C*2) symmetry, and flexibly tethered DNA-binding C-terminal domains that adopt two distinct positions relative to the N-terminal domains. Superposition of all 400 C^α^ atoms in common with the CAP dimer of PDB entry 1g6n (Passner *et al.*, 2000 ▶) yields a root-mean-square deviation (r.m.s.d.) of only 1.4 Å. The ‘closed’ conformation of subunit *A* (Fig. 1 ▶ *a*, blue/light-blue ribbon) closely resembles the conformation of DNA-bound CAP (Schultz *et al.*, 1991 ▶; Parkinson *et al.*, 1996 ▶). In the ‘open’ conformation of subunit *B* (Fig. 1 ▶ *a*, green/lime ribbon), the C-terminal domain is rotated by 40° and translated by 15 Å relative to the closed conformation.

The full *A* and *B* subunits superimpose poorly, owing to their observed conformational difference (r.m.s.d. of 3.9 Å). However, the individual N- and C-terminal domains from each subunit readily superimpose (r.m.s.d.s of 1.1 and 0.4 Å, respectively). The N- and C-terminal domains of the new structure also readily superimpose with previously determined cAMP–CAP crystal structure domains [r.m.s.d.s of ≤0.9 and ≤1.5 Å, respectively, from PDB entries 1g6n (Passner *et al.*, 2000 ▶), 4hzf (Rodgers *et al.*, 2013 ▶) and 2gzw (RIKEN Structural Genomics/Proteomics Initiative, unpublished work)].

The two Co^2+^ ions bound to the CAP dimer have chemically similar environments on each N-terminal domain, even though the positions are not crystallographically equivalent (Figs. 1 ▶ *a* and 1 ▶ *b*, purple spheres). Each has approximate octahedral coordination (Fig. 1 ▶ *b*), with three liganding atoms contributed by N-terminal domain residues His19 (N^∊2^), His21 (N^∊2^) and Glu96 (O^∊1^ or O^∊2^). In each case, a neighboring CAP dimer related by crystal symmetry contributes two additional ligands from residue Glu37 (O^∊1^ and O^∊2^). Octahedral coordination is completed at each site by a solvent water O atom. The average Co^2+^-to-coordinating-atom bond length is 2.22 ± 0.12 Å (12 measurements; range 2.03–2.44 Å) and the average atom-to-Co^2+^-to-neighbor-atom bond angle is 92.0 ± 11.0° (21 measurements; range 57.0–113.1°).

13 sulfate anions were also identified at positions adjacent to the protein surface (Fig. 1 ▶; sulfates are shown in stick representation with sulfur in yellow and oxygen in red). Six of these mediate protein–sulfate–protein contacts between the CAP dimer and crystal lattice neighbors (Table 2 ▶).

## 4. Discussion   

The identification of Co^2+^ tightly bound to the CAP AR2 region confirms the hypothesis originally put forward by Wickstrum & Egan (2002 ▶) that CAP can bind metals and, in addition, that residue His19 is involved in the metal-binding activity. Their hypothesis was based on the observation that CAP can effectively bind to Ni^2+^-charged chromatography media, with wild-type CAP eluting at a substantially higher concentration of imidazole than the CAP mutant His19Ala.

The three N-terminal domain Co^2+^-binding residues (His19, His21 and Glu96) constitute a major subset of the four residues previously identified to form CAP activating region 2 (AR2), which participates in transcription activation specifically at class II CAP-dependent promoters (Niu *et al.*, 1996 ▶). *In vivo*, His19Ala, His21Ala and Lys101Ala mutations each result in an approximately fivefold reduction in class II transcription activation, while Glu96Ala mutation results in an approximately threefold improvement; these same mutations have no effect on class I transcription activation (Niu *et al.*, 1996 ▶). At class II promoters, CAP binds to a site ∼−45 bp upstream of the transcription start site, and CAP-AR2 is predicted based on cross-linking analysis to contact a highly acidic loop in the N-terminal domain of the RNAP α^I^ subunit with sequence EEDE (residues 162–165; Niu *et al.*, 1996 ▶; Lawson *et al.*, 2004 ▶).

In the crystal structure reported here, Co^2+^-metal-binding coordination by the three AR2 residues is completed through an acidic residue provided in ‘*trans*’ through a crystal contact. Although no specific metal requirement has been reported for CAP class II CAP-dependent transcription activation, our structure raises the possibility that the local environment of AR2 in contact with the acidic loop of the RNAP α^I^ subunit could involve or accommodate metal binding.

Several sulfates in the crystal structure adopt positions that correspond to phosphate sites of the DNA backbone in CAP–DNA crystals (Table 2 ▶, Fig. 1 ▶ *c*). The DNA in the CAP dimer–DNA complexes is strongly bent and electrostatic interactions are thought to play an important role in DNA bending (Schultz *et al.*, 1991 ▶; Gartenberg & Crothers, 1988 ▶; Kapanidis *et al.*, 2001 ▶); our structure suggests that the CAP C-terminal domains have an intrinsic capability to bind to DNA half-sites prior to adoption by CAP of its fully bent DNA binding conformation.

## Supplementary Material

PDB reference: catabolite activator protein, 4ft8

## Figures and Tables

**Table 1:** Crystal, data collection and refinement statistics and model information Values in parentheses are for the outer resolution shell.

Crystal data	
Crystal system, space group	Orthorhombic, *P*2_1_2_1_2
Unit-cell parameters (Å)	*a* = 104.14, *b* = 108.45, *c* = 44.32
No. of CAP dimers in unit cell *Z*	4
Matthews coefficient *V*_M_ (Å^3^ Da^−1^)	2.50
Solvent content (%)	50.8
Data collection	
Diffraction source	Beamline X8C, NSLS
Wavelength (Å)	1.1
Detector	ADSC Quantum 4
Temperature (°C)	−173
Resolution range (Å)	50.0–1.97 (2.03–1.97)
No. of unique reflections	35645 (3280)
No. of observed reflections	181462
Completeness (%)	97.1
Multiplicity	5.1 (4.2)
〈*I*/σ(*I*)〉	9.5
*R*_merge_	0.088 (0.778)
Data-processing software	*DENZO*, *SCALEPACK*
Refinement	
Refinement software	*phenix.refine*, *PHENIX*
σ cutoff	*F* > 1.340σ(*F*)
Resolution range (Å)	40.78–1.97 (2.02–1.97)
No. of reflections used in refinement	33823
No. of reflections above σ cutoff	35597
Final overall *R* factor	0.194
Atomic displacement model	Individual isotropic *B* factors
Overall average *B* factor (Å^2^)	35.5
No. of protein atoms	3268
R.m.s.d., bond lengths (Å)	0.010
R.m.s.d., bond angles (°)	1.007
No. of ligand atoms	116
No. of solvent atoms	410
Total No. of atoms	3794
No. of refined parameters	15176
NCS restraints	Removed in final refinement cycles
Bulk-solvent model	Flat; *B*_sol_ = 58.65 Å^2^, *k*_sol_ = 0.37 e Å^−3^
Final *R*_work_	0.194 (0.286)
No. of reflections for *R*_free_	1774
Final *R*_free_	0.229 (0.324)

**Table 2:** cAMP–CAP dimer sulfate environments

Crystal asymmetric unit environment	Crystal lattice neighbors	CAP region
His21*A* N^δ1^, Lys22*A* N	Lys35*B* N^ζ^	AR2
His21*B* N^δ1^, Lys22*B* N	Lys35*A* N^ζ^	AR2
Ile20*A* N		AR2
Asp68*B* O^δ1^, Gln119*B* O^∊1^, Arg122*B* N^∊^,N^η2^, Arg123*B* N^∊^,N^η2^		cAMP site
His31*A* N^δ1^, Trp85A N^∊1^	His31*B* N^δ1^, Trp85B N^∊1^	cAMP site
Glu81*A* O^∊2^	Thr28*B* N	cAMP site
Ala91*A* N		
Val139*B* N, Arg142*B* N^η1^		DNA PO_4_
Arg169*A* N, Gln170*A* N		DNA PO_4_
Thr168*B* O^γ1^, Arg169*B* N, Gln170*B* N		DNA PO_4_
Lys57*B* Nζ, Ser 179*B* N,O^γ^, Thr182*B* O^γ1^		DNA PO_4_
Arg180*B* N,N^η1^,N^η2^		DNA binding
Lys201*B* N	Ty 206*A* O^η^	DNA PO_4_
